# Editorial of non-human primate research

**DOI:** 10.1093/nsr/nwad326

**Published:** 2023-12-21

**Authors:** Mu-ming Poo

**Affiliations:** Scientific Director, CAS Center for Excellence for Brain Science and Intelligence Technology, China

The progress of biology and medicine over the past century has been greatly facilitated by research based on animal models that could be used for experimentation. Small animal models such as the nematode worm *C. elegans* and the fruit fly *Drosophila* played crucial roles in the development of genetics, embryology, and cell biology. The mouse has become the quintessential animal model in the past few decades, largely due to development of mouse lines that have acquired genetic homogeneity via inbreeding over many generations, and many transgenic mouse lines carrying modified genetic makeup are also available. Mouse lines mimicking a variety of human diseases caused by genetic mutations are widely used for studying pathogenic mechanisms and for testing the efficacy of therapeutic treatments prior to human clinical trials.

In this issue of *NSR*, the Special Topic on ‘Non-human Primates as Animal Model Systems’ focuses on two animal species that are closest to humans, the macaque and marmoset monkeys, whose evolutionary lineages were separated from the human lineage about 25 and 35 million years ago, respectively. These two types of monkeys represent the principal non-human primate (NHP) models currently used by the scientific community. Articles included in this Special Topic illustrate various research directions in basic biology and biomedical sciences. Due to the existence of primate-specific biological and physiological functions, the use of NHPs as animal models is essential for biomedical sciences. Nevertheless, non-primate animal models remain highly useful for studying mechanisms and functions that that are conserved between primate and non-primate species.

As articulated in the review by Runrui Zhang, Fucheng Luo and colleagues [1] on the neurogenesis in primates versus rodents, comparative studies of neurogenesis across rodents, NHPs, and humans clearly point to both conservation and divergence of cellular and molecular features among species. A prominent primate-unique process of neurogenesis is the appearance of a new type of highly proliferative neuroprogenitor, known as the outer radial glial cells, that give rise to a large population of cortical cells, thickening of the neocortex, and the emergence of cortical folds. There is also the expression of primate-specific genes related to the delay in the pace of brain development, a phenomenon known as neoteny. The neoteny of brain development allows more extensive growth of the brain tissue and formation of more complex neural connections, leading to higher cognitive functions of the primate brain, such as complex decision-making, empathy, self-awareness, prosocial behaviors, and complex vocal communication.

One approach to delineate the function of human-specific genes is to introduce them into monkeys and examine the effect of the transgenes on the structure and function of the brain. In a research article in this issue, Bing Su, Cirong Liu and colleagues [2] generated transgenic cynomolgus macaque monkeys carrying the human-specific *SRGAP2C* gene. Structural studies using magnetic resonance imaging of the developing monkeys revealed that this transgene delayed brain development with region-specific volume changes, increased axon myelination in temporal and occipital cortices and the number of deep-layer neurons during neurogenesis, as well as delayed synaptic maturation in the adolescence period. Global analysis of the gene expression profiles by single-cell mRNA sequencing also revealed the neotony at the molecular and cellular levels, in terms of the molecular pathways related to neuron ensheathment, synaptic connections, extracellular matrix, and energy metabolism. Importantly, the authors demonstrated that these monkeys had improved motor planning and execution skills. This study, together with the authors’ previous work on monkeys expressing another human gene *MCPH1* [3], could explain at least in part how the emergence of human-specific genes could result in the neotony in human brain development and enhanced cognitive functions.

Among all primate species, humans are unique in their ability to communicate by using language with syntax, grammar, and open-ended construction of sentences. Efforts over many decades by primatologists and psychologists in teaching NHPs (particularly chimpanzees) to learn human language have failed. It is generally assumed that experience-dependent formation of human-specific neural circuits during brain development is required for human language acquisition. One approach to understand the evolutionary origin of this capability is to study vocal communication in marmosets, which exhibit complex prosocial behaviors and a rich repertoire of vocal sounds for communication. Studying neural circuits underlying vocalization and the perception of vocal sounds in marmosets may reveal some clues to the evolution of language in humans.

Central processing of sounds begins at the primary auditory cortex, followed by transmission of auditory signals to higher cortical areas involved in more complex signal integration and perception. In addition to the cortices, subcortical areas are likely to play an important role in processing emotional aspects of vocalization. By recording single neuron activities in awake marmosets, Lixia Gao, Xinjian Li and colleagues reported in this issue [4] that many neurons in the amygdala, a subcortical nucleus known to be involved in processing emotion, could respond selectively to calls made by the same marmoset species as well as calls by specific individual marmosets. These findings point to the amygdala as an area involved in socially relevant perceptual processing of call signals.

Actions of human-specific genes not only endow the human brain with unique structure and function but also brain disorders when the expression of these genes becomes abnormal. The causes of most psychiatric diseases are multifaceted, including inherited mutations of many risk genes and their regulatory elements in the DNA and abnormal epigenetic modifications during embryonic development and after birth. An example is the obsessive-compulsive disorder (OCD) that causes compulsive ritualistic behaviors in patients. Zhen Wang, Zhiqi Xiong, Rongwei Zhai and their colleague [5] reported in this issue on their discovery of a small group of rhesus monkeys from large monkey colonies in China that exhibited ritualistic behaviors resembling OCD symptoms—persistent sequential motor behaviors in individual-specific sequences. These monkeys also showed abnormality in the size of specific brain regions related to motor and cognitive actions and in the corpus callosum, the large nerve bundle connecting the two brain hemispheres. They also showed some mutations of genes known to be risk genes for OCD in humans, and the anti-psychosis drug fluoxetine for human OCD patients also reduced the OCD-like monkey behaviors. This spontaneously occurring monkey model of OCD may become useful in screening new drugs and neuromodulation treatments for OCD in the future.

There is a long tradition in neuroscience in using rhesus monkeys to study a wide range of cognitive functions, such as sensory processing and perception, multisensory integration, decision-making based on accumulated evidence, motor planning and execution, as well as consciousness. Experimental tools initially developed for monkey studies is now beginning to be applied in biomedical studies. In this issue, Yong Gu, Jijun Wang and colleagues [6] designed a sensitive high-dimensional method for monitoring eye movements in rhesus monkeys that could measure multiple oculomotor parameters for saccade, smooth pursuit, and fixation of monkey eyes. Two commonly used psychoactive drugs were also shown to produce large reductions in the accuracy and increment in reaction time as well as in pupillary responses during monkey's performance of high cognitive-demanding tasks. Notably, similar abnormalities of eye movements were also found in patients with schizophrenia and a machine learning algorithm could reliably identify altered states induced in monkeys by the two drugs with very high accuracy. These findings showed that this rhesus monkey model could be used for future screening of the efficacy of new drugs for the treatment of psychosis.

The five articles in the Special Topic of this issue represent only a glimpse of the current NHP research, an area that has grown rapidly in China during the past decade. This is testified by a wide range of research articles on NHPs published by *NSR* in recent years. The topics ranged from transcriptomic profiling of aging NHP tissues [7,8], synaptic structure dynamics in the adult monkey brain [9], syllable-selective neurons in the primary auditory cortex of marmosets [10], generation of a monkey model of psychiatric diseases by deletion of the core gene *BMAL1* for circadian rhythm [11], cloning of a *BMAL1*-deleted monkey by somatic cell nucleus transfer [12], accelerated generation of macaque monkeys [13], and a monkey showing a spontaneous Parkinson-like phenotype [14]. As elaborated by two commentaries in the Critique and Debate section of *NSR* [15,16] and a review on genome editing and stem cells in NHPs [17], there is strong justification and increasing consensus on the need for further developing NHP animal models for both basic biology and biomedical sciences. In particular, NHP models of brain diseases appear to be indispensable for effective preclinical studies of candidate drugs and physiological neuromodulation.

***Conflict of interest statement**.* None declared.
